# Replication and Transcription Activities of Ribonucleoprotein Complexes Reconstituted from Avian H5N1, H1N1pdm09 and H3N2 Influenza A Viruses

**DOI:** 10.1371/journal.pone.0065038

**Published:** 2013-06-04

**Authors:** Karry L. K. Ngai, Martin C. W. Chan, Paul K. S. Chan

**Affiliations:** 1 Department of Microbiology, Faculty of Medicine, The Chinese University of Hong Kong, New Territories, Hong Kong Special Administration Region, People’s Republic of China; 2 Department of Microbiology and Stanley Ho Centre for Emerging Infectious Diseases, Faculty of Medicine, The Chinese University of Hong Kong, New Territories, Hong Kong Special Administration Region, People’s Republic of China; University of Malaya, Malaysia

## Abstract

Avian influenza viruses pose a serious pandemic threat to humans. Better knowledge on cross-species adaptation is important. This study examined the replication and transcription efficiency of ribonucleoprotein complexes reconstituted by plasmid co-transfection between H5N1, H1N1pdm09 and H3N2 influenza A viruses, and to identify mutations in the RNA polymerase subunit that affect human adaptation. Viral RNA polymerase subunits PB1, PB2, PA and NP derived from influenza viruses were co-expressed with pPolI-vNP-Luc in human cells, and with its function evaluated by luciferase reporter assay. A quantitative RT-PCR was used to measure vRNA, cRNA, and mRNA levels for assessing the replication and transcription efficiency. Mutations in polymerase subunit were created to identify signature of increased human adaptability. H5N1 ribonucleoprotein complexes incorporated with PB2 derived from H1N1pdm09 and H3N2 viruses increased the polymerase activity in human cells. Furthermore, single amino acid substitutions at PB2 of H5N1 could affect polymerase activity in a temperature-dependent manner. By using a highly sensitive quantitative reverse transcription-polymerase chain reaction, an obvious enhancement in replication and transcription activities of ribonucleoproteins was observed by the introduction of lysine at residue 627 in the H5N1 PB2 subunit. Although less strongly in polymerase activity, E158G mutation appeared to alter the accumulation of H5N1 RNA levels in a temperature-dependent manner, suggesting a temperature-dependent mechanism in regulating transcription and replication exists. H5N1 viruses can adapt to humans either by acquisition of PB2 from circulating human-adapted viruses through reassortment, or by mutations at critical sites in PB2. This information may help to predict the pandemic potential of newly emerged influenza strains, and provide a scientific basis for stepping up surveillance measures and vaccine production.

## Introduction

Influenza A virus contains eight single-stranded RNA segments. The negative-sense viral RNA (vRNA) segments act as templates for messenger RNA (mRNA) synthesis in transcription, and for complementary RNA (cRNA) synthesis which is used for replication of vRNA. Both transcription and replication are performed by viral RNA-dependent RNA polymerase (RdRp) inside the nucleus of infected cells [1], [2]. The RdRp complex composed of three polymerase subunits, polymerase basic protein 1 (PB1), polymerase basic protein 2 (PB2) and polymerase acid protein (PA). These proteins, in association with nucleoproteins (NP) and vRNA segments, constitute viral ribonucleoproteins (RNPs) [3]. The PB1 subunit contains the conserved motif of RdRp [4], [5] and is implicated in promoter binding [6]. The PB2 subunit binds to the cap of host mRNA to generate capped RNA primers for the initiation of mRNA synthesis [7], [8]. Although the role of PA remains uncertain, it has been suggested to function in both transcription and replication mediated by its endonuclease [1], [9], [10], [11], and binding to vRNA promoter and cap [10].

Since 1997, sporadic human infections with highly pathogenic H5N1 viruses have been reported. Although an efficient and sustained transmission of highly pathogenic H5N1 viruses in humans has yet occurred [12], [13], their capability to overcome species barrier and adapt to human infection remains a serious threat. Over the past century, four influenza pandemics have occurred. Pandemic strains of H2N2 in 1957, and H3N2 in 1968 were created by reassortments resulting in the acquisition of an avian polymerase base protein 1 (PB1) gene segment [14]. The H1N1pdm09 virus was a triple reassortant containing gene segments from human, avian, and swine influenza viruses [15]. The H1N1pdm09 virus retained the “human” PB1 gene that was descended from an avian virus in 1968, and it had also acquired avian PA and PB2 genes.

It is clear that preferential binding of haemagglutinin (HA) to terminal α-2,3 and α-2,6-linked sialic acid receptors on host cell surface is not the sole barrier of cross-species infection [16]. Previous work has shown that RNP complex is one of the key determinants in host selection, adaptation and pathogenicity of avian viruses [2], [17]. RNP complex has multiple functions including viral transcription and replication, interaction with cellular host factors; and all these functions must be efficiently carried out in humans for a novel pandemic strain to emerge [18], [19]. We hypothesized that functional activity of H5N1 RNP complex in human cells was limited by its subunits of avian origin. Here, we examined the transcription and replication efficiency of RNP complexes reconstituted from different combinations of PB1, PB2, PA, and NP derived from avian H5N1, H1N1pdm09 and H3N2 influenza A viruses.

## Methods

### Cell Culture and Virus Strains

Human embryonic kidney 293T cells (ATCC, CRL-11268) were used as an *in-vitro* model to examine the polymerase activity of viral RNP complexes. Cells were maintained in Dulbecco’s modified Eagle’s medium (DMEM) supplemented with 10% fetal bovine serum (FBS) (Life Technology, Rockville, MD) at 33°C or 37°C in a 5% CO_2_ incubator.

cDNA clones originated from four influenza virus strains were used to generate different RNP complexes. These viruses include: A/Thailand/1(KAN-1)/2004 (H5N1), representing highly pathogenic influenza A H5N1 viruses; A/HongKong/CUHK-72079/2009 (H3N2), representing seasonal H3N2 viruses; and A/Auckland/1/2009, representing the 2009 pandemic virus (H1N1pdm09). The A/WSN/33 (WSN) H1N1 virus was used as a reference strain in this study.

### Expression of Recombinant Viral RNPs in Transfected Cells

The protein expression plasmids for three polymerase subunits (PA, PB1 and PB2) and nucleoprotein (NP) of H1N1pdm09 and H3N2 had been described previously [20]. The expression plasmids of WSN H1N1, pPolI-vNP-Luc and pPolI-NA were kindly provided by Prof. George Brownlee [1], [21]. The full-length PA, PB1, PB2 and NP genes of H5N1 were PCR amplified using Fusion polymerase (Stratagene, La Jolla, CA). PB1 and PB2 were inserted into expression plasmid pcDNA3A using *Hind*III and *Not*I restriction sites, whereas the *BamH*I and *Not*I restriction sites and the *Kpn*I and *Not*I restriction sites were used for PA and NP, respectively. Point mutations were introduced into the PB2 plasmid of H5N1 to generate three different mutants with the following nucleotide substitutions: (i) 473, A→G causing E158G, (ii) 811, A→G causing T271A, (iii) 1879, G→A causing E627K. The identities of these clones were confirmed by sequencing.

Various combinations of PA, PB1, PB2 and NP-expression plasmids derived from different subtypes of influenza virus were used to generate hybrid viral RNP complexes. 293T cells in 48-well plates were co-transfected with 0.4 µg of each plasmid and pPolI-NA by using Lipofectamine 2000 (Invitrogen, Carlsbad, CA) for 48 hrs at 33°C or 37°C.

### Luciferase Reporter Assay for Viral Polymerase Activities

Luciferase reporter assay was performed as described before [22]. In brief, 50 ng of each plasmid and pPolI-vNP-Luc were co-transfected to generate viral polymerase complexes in 96-well plates. At 48 hours post-transfection, luciferase production was measured by Dual-Glo Luciferase Assay System (Promega, Madison, WI) according to the manufacturer’s instructions. Polymerase activity was normalized with the expression of a reporter plasmid pGL4.73 [hRluc/SV40] (Promega), encoding a *Renilla* luciferase gene.

### RNA Extraction and cDNA Synthesis

Total RNA was extracted from transfected cells using TRIzol plus RNA purification kit (Invitrogen) and followed by DNaseI treatment (New England Biolabs, Ipswich, MA, USA). A previously described approach was used to achieve specific quantification for each RNA species [23], [24]. Briefly, separate aliquots of the extracted RNA preparations were used for reverse transcription and subsequently for real-time PCR. Specific reverse transcription of mRNA was achieved by using oligo-dT (50 pmol, 50°C), whereas specific reverse transcription of vRNA and cRNA was achieved by primer 5'-AGC AAA AGC AGG G-3' (10 pmol, 55°C), and primer 5'-AGT AGA AAC AAG G-3' (10 pmol, 55°C), respectively. Reverse transcription reaction was carried out with SuperScript III reverse transcriptase (Invitrogen). cDNA synthesis was performed using 160 pg of extracted total RNA at the indicated temperature for 60 min and then heat inactivated at 70°C for 20 min. RNA and cDNA were stored at −70°C until further use.

### Quantitative RT-PCR Assays

Quantitative reverse transcription-polymerase chain reaction (qRT-PCR) was used to quantify mRNA, vRNA and cRNA levels using Power SYBR® Green PCR Master Mix (*Applied Biosystems*, Foster City, CA). Five microlitres of the corresponding cDNA sample were added to the 25-µL reaction. Primers were 5'-CCG GCA AAG TGA TGT GTG TGT G-3' (corresponding to nucleotide 806 to 827 of NA cRNA) and 5'-CCG AAA ACC CCA CTG CAG ATG-3' (complementary to nucleotide 900 to 920 of NA cRNA). Reactions were first incubated at 50°C for 2 min, followed by 95°C for 10 min, and subjected to 40 cycles of 95°C for 15 s and 60°C for 1 min. The concentration of viral RNA were normalized with the corresponding input 5 S rRNA by primer 5′- TAC GGC CA TAC CAC CCT GAA C -3′ and primer 5′- CGG TAT TCC CAG GCG GTC T -3′.

### Statistical Analysis

All data were generated from three independent experiments. The polymerase activities and viral RNA levels were quantified and compared with values obtained from the corresponding parental viral RNP complex which was set as 100%. The activity of replication was reflected by cRNA and vRNA levels, while the activity of transcription was reflected by mRNA level. Differences in the normalized ratios of various viral RNP complexes were compared by the Student’s *t*-test. *P*-values less than 0.05 were regarded as significant.

## Results

### Polymerase Activity of Parental H1N1pdm09, H3N2, H5N1 and WSN H1N1 at 33°C and 37°C

To characterize the effect of temperature on avian and human influenza viruses, we compared the polymerase activities of parental H5N1, H1N1pdm09 and H3N2 RNP complexes in human cells under incubation at 33°C and 37°C, mimicking physiological temperatures of human upper and lower respiratory tracts, respectively. H1N1pdm09 showed a significantly higher activity at 33°C than 37°C (relative activity: 13.8 vs. 6.16, *P* = 0.006); H3N2 showed no significant difference between 33°C and 37°C (relative activity: 7.0 vs. 5.5, *P* = 0.49); whereas H5N1 showed a significantly higher activity at 37°C (relative activity: 0.3 vs. 1.2, *P*<0.001).

**Figure 1:**
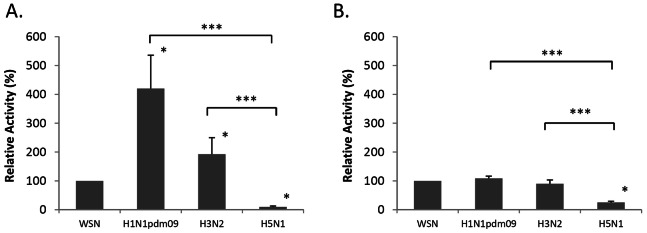
Comparison of *in vitro* polymerase activity of H1N1pdm09, seasonal H3N2, avian H5N1, and WSN H1N1. 293T cells were co-transfected with expression plasmids of NP, PA, PB1 and PB2 together with pPolI-vNP-Luc and a reporter plasmid pGL4.73 [hRluc/SV40], encoding a *Renilla* luciferase gene. Cells were incubated at (A) 33^o^ and (B) 37°C. Polymerase activity was normalized with the expression of a reporter plasmid. Relative polymerase activity (%) was expressed as relative activity to WSN H1N1 in percentage. Results shown are means with standard deviations from three independent assays. *indicates *P*<0.05 when compared to WSN H1N1, and ***indicates *P*<0.001 when compared to H5N1.

The polymerase activity was further analyzed with reference to WSN H1N1, which is a commonly used reference strain (Figure 1). At 33°C, H3N2 and H1N1pdm09 showed a significantly higher activity (193% and 420% of WSN H1N1; P = 0.042 and 0.003, respectively), but H5N1 had significantly lower activity compared to WSN H1N1 (10% of WSN H1N1, P<0.001). At 37°C, the polymerase activity of H3N2 and H1N1pdm09 was similar to WSN H1N1, whereas that of H5N1 was significantly lower than all others (25% of WSN H1N1, P<0.001).

### RNP Complexes Reconstituted from H5N1, H1N1pdm09 and H3N2

#### Effects of PB2 substitution

**Figure 2:**
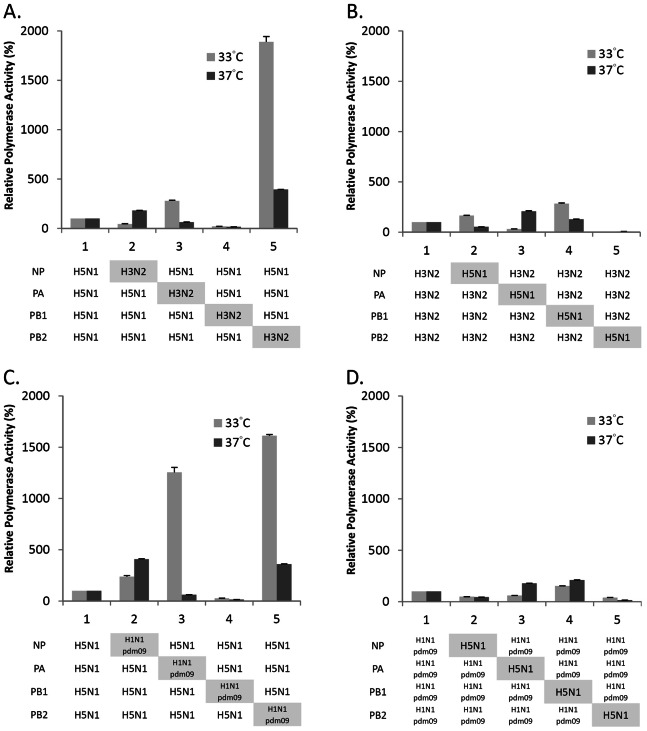
Comparison of *in vitro* polymerase activity of reconstituted RNP complexes. Activities of polymerase complexes with a single gene replacement following expression in human 293T cells. (A) H5N1 polymerase complexes substituted with one H3N2 gene, (B) H3N2 polymerase complexes substituted with one H5N1 gene, (C) H5N1 polymerase complexes substituted with one H1N1pdm09 gene, and (D) H1N1pdm09 polymerase complexes substituted with one H5N1 gene, were analyzed in 293T cells transfected with the indicated plasmids of NP, PA, PB1 and PB2 together with pPolI-vNP-Luc and a reporter plasmid pGL4.73. Cells were incubated at 33°C and 37°C. Polymerase activity was normalized with the expression of a reporter plasmid. Relative polymerase activity (%) was expressed as relative activity to the corresponding parental vRNPs. Results shown are means with standard deviations from three independent assays.

The polymerase activities of hybrid RNP complexes are shown in Figure 2. When the H5N1 PB2 was replaced by an H3N2 PB2 or H1N1pdm09 PB2, a significant increase in polymerase activity was observed. The effect was stronger at 33°C than 37°C (H3N2 PB1∶18.9-fold increase in activity at 33°C, *P*<0.001, and 4-fold at 37°C, *P*<0.001; H1N1pdm09 PB1∶16.1-fold at 33°C, *P*<0.001, and 3.6-fold at 37°C, *P*<0.001) (Figures 2A and 2C). In line with this, when the H3N2 PB2 or the H1N1pdm09 PB2 was replaced by H5N1 PB2, a significant decrease in polymerase activity was observed (Figure 2B and 2D).

#### Effects of PB1 substitution

When the PB1 subunit of H3N2 or H1N1pdm09 RNP complex was substituted with H5N1 PB1, a significant increase in polymerase activity was observed. The effect on H3N2 was stronger at 33°C (2.8-fold, *P* = 0.002) compared to 37°C (1.3-fold, *P*<0.001) (Figure 2B). In contrast, the effect on H1N1pdm09 was stronger at 37°C (2.1-fold, *P*<0.001) than at 33°C (1.5-fold, *P*<0.001) (Figure 2D).

When the H5N1 PB1 was replaced by either H3N2 or H1N1pdm09 PB1, a decrease in polymerase activity was observed at both 33°C and 37°C (H3N2∶0.2-fold at 33°C and 37°C, *P*<0.001; H1N1pdm09∶0.3-fold at 33°C, *P*<0.001; 0.1-fold at 37°C, *P*<0.001) (Figure 2A and 2C).

#### Effects of PA substitution

When the PA subunit of H5N1 RNP complex was substituted with H1N1pdm09 PA, a significant increase in activity was observed at 33°C (12.6-fold, *P* = 0.002), but not at 37°C (Figure 2C). A similar effect, but to a much smaller magnitude was observed when the H5N1 PA was replaced by H3N2 PA (Figure 2A).

On the other hand, when the H3N2 PA or H1N1pdm09 PA was replaced by H5N1 PA, a significant increase in polymerase activity was observed at 37°C (2.1-fold for H3N2, *P* = 0.002; 1.8-fold for H1N1pdm09, *P*<0.001), but a lower activity was observed at 33°C (Figures 2B and 2D).

#### Effects of NP substitution

Substitution with NP results in a relatively smaller effect on polymerase activity. When the H5N1 NP was replaced by H3N2 or H1N1pdm09 NP, a slight increase in polymerase activity at 37°C was observed (H3N2∶1.8-fold, *P*<0.001; H1N1pdm09∶4.1-fold, *P*<0.001) (Figures 2A and 2C).

On the other hand, when the H3N2 NP or H1N1pdm09 NP was replaced by H5N1 NP, a slight decrease in activity was observed at 37°C (Figures 2B and 2D).

#### Principal observations

Taken together, substitution of the H5N1 RNP complex with PB2 derived from either H3N2 or H1N1pdm09 resulted in a substantial increase in polymerase activity, and the effect was more pronounced at 33°C. Substitution of the H5N1 RNP complex with PA derived from H1N1pdm09 also achieved a pronounced increase in activity at 33°C. In contrast, substitution of the H5N1 RNP complex with PB1 derived either from H3N2 or H1N1pdm09 decreased the polymerase activity of H5N1.

### Effects of PB2 Mutations

**Figure 3:**
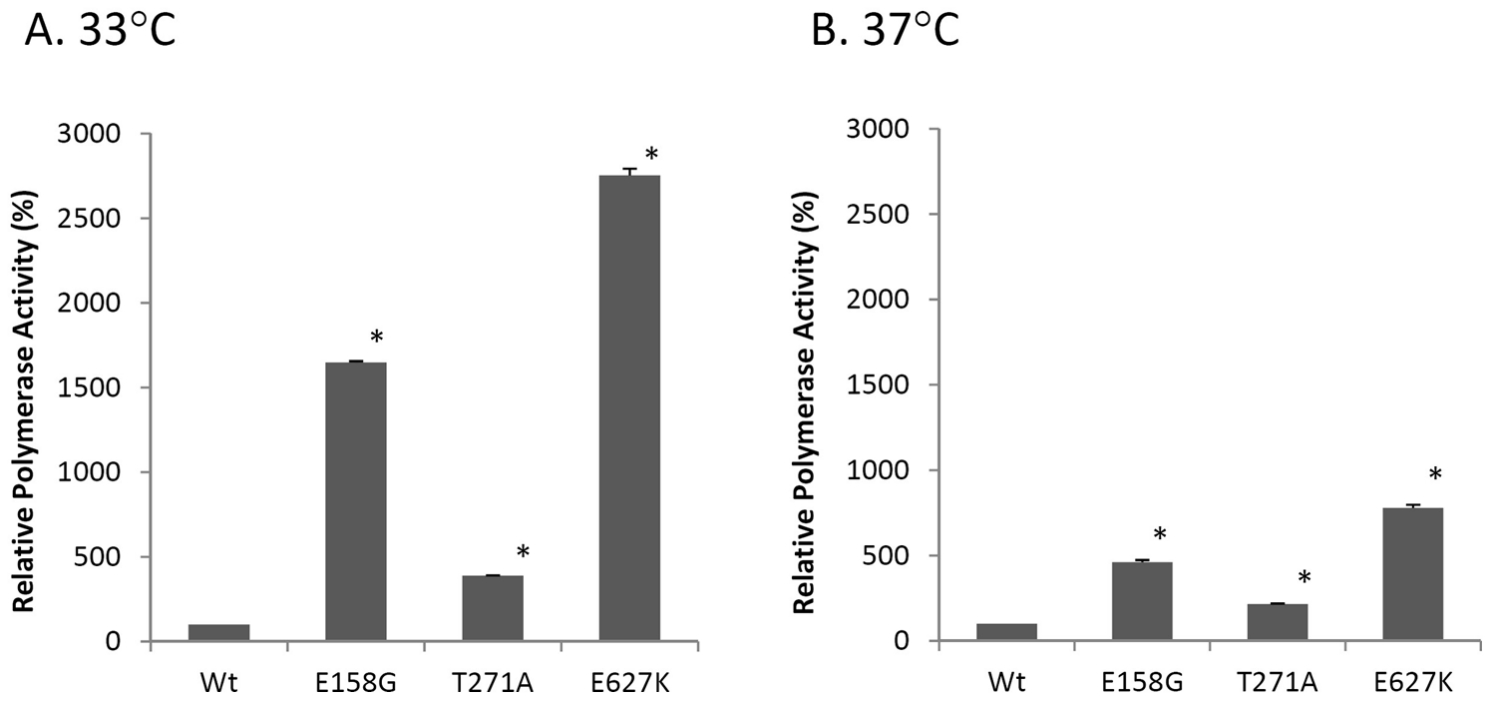
Polymerase activity of H5N1 RNP complexes containing mutations E158G, T271A and E627K in PB2. 293T cells were co-transfected with expression plasmids of NP, PA, PB1 and either wild type (WT) or PB2 mutants with the indicated amino acid substitution of E158G, T271A or E627K, together with pPolI-vNP-Luc and a reporter plasmid pGL4.73. Cells were incubated at (A) 33°C, (B) 37°C. Polymerase activity was normalized with the expression of a reporter plasmid. Relative polymerase activity (%) was expressed as relative activity to WT. Results shown are means with standard deviations from three independent assays. *indicates *P*<0.05 when compared to WT.

The effects of H5N1 PB2 mutations on polymerase activity are shown in Figure 3. At 33°C, E627K, E158G and T271A resulted in an increase in activity by 27.5-fold, 16.5-fold and 3.9-fold (*P*<0.001), respectively (Figure 3A). These mutations also led to an increase in activity at 37°C, though to a lesser extent compared to those at 33°C (Figure 3B). Among these mutations, E627K exhibited the most pronounced effect.

**Figure 4:**
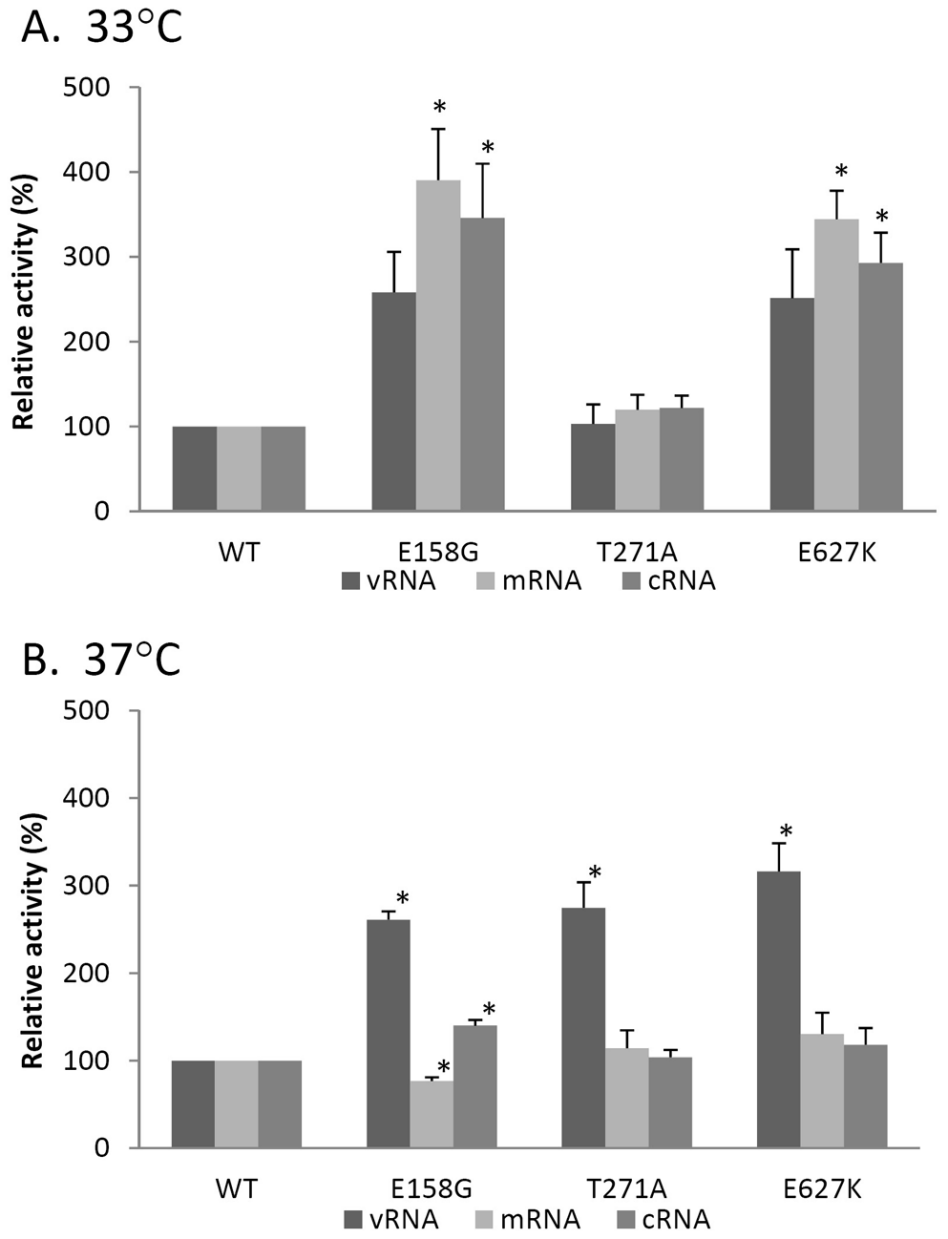
Quantitation of viral RNA levels of H5N1 RNP complexes containing PB2 mutations. 293T cells were co-transfected with expression plasmids of NP, PA, PB1 and either wild type (WT) of indicated PB2 mutants with amino acid substitution of E158G, T271A or E627K, together with pPolI-NA plasmid. Total cellular RNA was isolated after 48 hours post-transfection and subjected to quantitative RT-PCR for segment 6 (NA genes) transcripts. Cells were incubated at (A) 33°C, (B) 37°C. RNA levels were expressed as relative activity to wild-type. Results shown are means with standard deviations from three independent assays. *indicates *P*<0.05 when compared to wild-type.

We further investigated whether the higher activity associated with those mutations was linked to an increase in transcription (mRNA) and/or replication (cRNA and vRNA synthesis) by using a real-time PCR-based quantitative assay [18], [22]. A higher transcription activity of mutant complexes was observed at 33°C, where the mRNA levels of E158G and E627K RNP complexes were increased by 3.9-fold (*P* = 0.01) and 3.4-fold (*P* = 0.03), respectively (Figure 4). On the other hand, a higher replication activity was observed at 37°C, where the vRNA levels of E158G, T271A and E627K were significantly increased by 2.6-fold (*P*<0.001), 2.8-fold (*P* = 0.04) and 3.2-fold (*P* = 0.03), respectively. Among all the substitutions, E158G showed a stronger transcription activity compared to others at 33°C, whereas no differences among the three mutations were observed at 37°C.

## Discussion

The cross-species infection of influenza virus, probably of avian origin, has caused a serious pandemic in 1918 [25], [26]. Reassortment among H3N2, H1N1pdm09 and H5N1 viruses is a real concern. This study examined the polymerase activity of the hybrid RNP complexes of these viruses. As anticipated, RNPs of human influenza viruses, H3N2 and H1N1pdm09, were well adapted to 33°C which is more close to the temperature of human upper airway, but the activity of H5N1 was much lower at this temperature. This observation is in line with the fact that the current H5N1 viruses have limited replication in human upper airway, and thus they have low efficiency in transmission from human to human.

We found that the polymerase activity of H5N1 could be increased to a very large extent by substituting the RNP complex with a PB2 derived from either H3N2 or H1N1pdm09, and the effect was more pronounced at 33°C. This is an important concern as such reassortants may be able to adapt to human upper airway with increased human to human transmission efficiency. By contrast, mammalian PB1 abolished polymerase activity of H5N1-originated polymerase in human cells. These results clearly demonstrated that optimal polymerase activity, due to a better compatibility of avian PB1 and mammalian PB2, is probably required for efficient viral replication in human cells [27].

In addition to PB2, mutations in PA subunit have been reported to affect viral RNA replication [28]. Our results provided further evidence that the H1N1pdm09 PA dramatically enhanced viral polymerase activity of H5N1 at 33°C. Although the underlying mechanism of temperature dependency of polymerase activity is not clear, PA subunit is likely to be one of the keys in host range restriction.

The function of avian polymerase in human cells may be improved by substitution of H5N1 PB2 subunit with “human” residues. Previous studies have shown that 627K in PB2 enhanced polymerase activity [29] and facilitates efficient replication of avian viruses in human cells [30]. Others have shown that 701N in PB2 was associated with a high virulence due to enhanced interaction with importin α [18], [31]. We speculated that other residues in PB2 might also contribute to the adaptation of H5N1 in humans. In the current study, the H5N1 strain that we examined already had the 701N in the PB2 subunit. We used this strain to compare the effects of E158G, T271A and E627K mutations. These mutations were selected as they have previously been shown to enhance the viral polymerase activity of influenza H5N1 virus in mammalian cells [32], [33], [34]. Our data consolidated previous observations, and in addition, we found that the enhancing effect of E627K substitution was substantially stronger than those of E158G and T271A. Furthermore, we showed that the enhancing effect of E627K was more pronounced at 33°C, thus may favor adaptation to human upper respiratory tract and leading to efficient human to human transmission.

The differential quantitative analysis of different viral RNA species revealed that mutations E158G, T271A and E627K on PB2 enhanced both the transcription and replication activity of viral polymerase. However, notable differences in the profile of transcriptive (mRNA) and replicative (vRNA and cRNA) intermediates were observed between 33°C and 37°C. Similar to earlier study, the K627E mutation significantly reduced vRNA and cRNA promoter binding activities of PB2 in avian H5N1 virus [35]. At 37°C, all three mutations only resulted in the elevation of vRNA level but not mRNA and cRNA levels, suggesting that these mutations may only enhance the polymerase activity of cRNA-dependent vRNA synthesis but not the activity of vRNA-dependent mRNA and cRNA synthesis. In contrast, at 33°C, the effect on transcription was stronger than replication. Of note, the mutation E158G in PB2 showed a dramatically increase in mRNA level at 33°C, whereas the increase in levels of cRNA and vRNA was less pronounced.

The mechanism behind how mutations and subunits of different species origin influence the transcriptional and replication activity of RNP complex remains elusive. One possibility is differential thermal stability of RNP complex with positive and negative strand template RNA at restrictive and permissive temperatures, as suggested in seasonal H1N1 virus [36]. Alternatively, it is known that RNP complex interact with a multitude of host intracellular proteins, including MCM and hCLE with PA, RanBP5 and Ebp1 with PB1, and RAF-1/Hsp90 with PB2 [37]. These cellular proteins has diverse effect on RNP complex. For example, Ebp1 is a selective inhibitor of RNP complex [38] whilst RanBP5 is involved in the trafficking of RNP subunits into cell nucleus [39]. It is possible that avian-like and human-like RNP subunits interact differently with these host factors. Further investigation is warranted.

### Conclusions

In conclusion, substitution of H5N1 RNP complexes with subunits, especially PB2, derived from H3N2 or H1N1pdm09 viruses could remarkably increase its replication and transcription activity in human cells. This indicates that some residues in human PB2 subunit might be involved in human adaptation. By using a highly sensitive quantitative RT-PCR, consistent with the result of polymerase activity, an obvious enhancement in replication and transcription activity of RNPs was observed by introduction of lysine at residue 627 in PB2 subunit. Although less strongly in polymerase activity, the temperature dependency of E158G mutation appeared to alter the accumulation of viral RNA levels, suggesting a temperature-dependent mechanism in regulating transcription and replication exists.
